# Investigation of the enzyme-inhibitory, antibacterial, and anticancer properties of metal phthalocyanines

**DOI:** 10.55730/1300-0527.3788

**Published:** 2026-02-16

**Authors:** Meryem TOPAL, Fevzi TOPAL, Fırat YILMAZ, Esra BULUT ATALAY, Ümit Muhammet KOÇYİĞİT, Emre GÜZEL

**Affiliations:** 1 Vocational School of Health Services, Department of Medical Services and Techniques, Gumushane University, Gümüşhane, Turkiye; 2 Department of Nutrition and Dietetics, Faculty of Health Sciences, Gumushane University, Gümüşhane, Turkiye; 3 Department of Food Engineering, Faculty of Engineering and Natural Sciences, Gumushane University, Gümüşhane, Turkiye; 4 Department of Biochemistry, Faculty of Science, Sivas Cumhuriyet University, Sivas, Turkiye; 5 Department of Biochemistry, Faculty of Pharmacy, Sivas Cumhuriyet University, Sivas, Turkiye; 6 Department of Engineering Fundamental Sciences, Faculty of Technology, Sakarya University of Applied Sciences, Sakarya, Turkiye

**Keywords:** Acetylcholinesterase, antibacterial activity, anticancer activity, butyrylcholinesterase, phthalocyanine

## Abstract

In this study, the enzyme inhibitory, antibacterial, and anticancer activities of a phthalonitrile derivative and its metal phthalocyanines (Pcs; indium, zinc, copper, cobalt, and manganese; 1–6) were investigated. The cobalt and manganese Pcs were synthesized for the first time in this study. The inhibitory activities of the symmetric Pcs, which were expected to interact with acetylcholinesterase (AChE) and butyrylcholinesterase (BChE) via their 4-(isopropylbenzyl)oxy substituents through host–guest interactions, were investigated. More selective inhibition of AChE than BChE was observed for these compounds. For AChE, CoPc was identified as the most potent inhibitor (IC_50_ = 14.81 nM), whereas for BChE, InPc was identified as the most potent inhibitor (IC_50_ = 56.35 nM). The K_i_ values indicated that most compounds exhibited competitive inhibition; copper phthalocyanine (CuPc) showed particularly strong inhibition against AChE (K_i_ = 3.08 nM **±** 1.12), whereas the lowest K_i_ value against BChE was observed for MnPc (K_i_ = 25.98 ± 1.97 nM). Most compounds exhibited competitive inhibition; however, CuPc showed competitive inhibition toward AChE but a noncompetitive inhibition pattern toward BChE. Dual inhibition of AChE and BChE by these compounds may be promising for addressing cholinergic deficits associated with Alzheimer’s disease. In addition, the acceptability of compounds 1–6 with respect to pharmacological drug-likeness criteria was assessed based on predicted absorption, distribution, metabolism, excretion (ADME) outcomes. In antibacterial tests, varying levels of inhibition were observed against selected bacterial strains. In anticancer assays, all compounds exhibited high cytotoxicity against the MCF-7 breast cancer cell line. Higher antiproliferative activity was observed for CuPc than for the other compounds. Morphological changes were induced in cancer cells by CuPc and MnPc. Overall, these compounds may have potential as enzyme inhibitors and as antibacterial and anticancer agents.

## 1. Introduction

Alzheimer’s disease (AD) is a prevalent form of dementia and is characterized as a chronic neurodegenerative disorder with complex, multifactorial causes. Neural tissue function and integrity are disrupted across multiple brain regions in this condition, ultimately leading to marked cognitive decline in advanced stages [1]. Acetylcholinesterase (AChE; EC 3.1.1.7) is a cholinesterase-family enzyme that hydrolyzes acetylcholine (ACh) and plays a crucial role in cholinergic neurotransmission. It is particularly abundant in the brain, nerves, and red blood cells. A reduction in AChE activity may lead to neurological disorders and, in severe cases, death. Acetylcholine is the primary substrate of this enzyme [2,3]. A sudden decrease in acetylcholine levels may be fatal, whereas a gradual decline may contribute to AD [4]. The human brain contains approximately 100 billion neurons, and AD is characterized by neurodegenerative processes that involve damage to, or loss of, these cells [5]. Butyrylcholinesterase (BChE) is another enzyme that is widely distributed in organs such as the lungs, brain, heart, liver, muscles, and kidneys and is also present in body fluids, including serum, sweat, and cerebrospinal fluid. Because BChE is synthesized primarily in the liver, it has been used as a clinical marker of liver function. Both AChE and BChE are implicated in Alzheimer’s disease, which is characterized by symptoms such as cognitive impairment, memory loss, and personality changes [6–8].

Phthalocyanines (Pcs) are a class of synthetic dyes widely used in various industries due to their unique optical and electronic properties [9]. Due to their unique chemical structures and photophysical properties, Pcs have attracted considerable attention across diverse fields, including medicinal chemistry. Pcs constitute a versatile class of compounds that has been associated with diverse biological activities, including anticancer, antimicrobial, and enzyme-inhibitory effects. Their potential as anticancer agents, antimicrobial agents, and enzyme inhibitors has been extensively studied [10–12]. Promising antimicrobial activity has been reported for Pcs against diverse pathogens, including bacteria, fungi, and viruses. Some Pcs have been reported to exhibit intrinsic antimicrobial activity, which may involve disruption of microbial cell membranes and interference with essential metabolic processes. With respect to enzyme inhibition, coordination with metal ions may enable Pcs to act as inhibitors of various enzymes, including oxidases and hydrolases. Binding of Pc substituents to enzyme active sites may block substrate access and/or alter conformational dynamics, thereby reducing catalytic activity. Inhibition of enzymes such as cholinesterases, which are relevant to Alzheimer’s disease, has been investigated [13]. Selectivity toward specific enzyme targets may be modulated through structural modification of Pcs, thereby providing a rationale for the development of novel therapeutic agents. Their mechanisms of action, particularly those involving light activation, may expand their potential applications in medical science. In this study, the Pc framework was designed based on a synergistic relationship among the macrocyclic core, peripheral substituents, and the central metal ion. The 4-(isopropylbenzyl)oxy group was selected as a peripheral substituent to serve two purposes: to mitigate the inherent tendency of Pcs to undergo π–π stacking and to enhance biological affinity. Pcs are known to undergo aggregation in solution, which can diminish solubility and reduce biological efficacy by decreasing the available surface area for molecular interactions [14]. The bulky 4-(isopropylbenzyl)oxy moiety may provide steric hindrance that reduces aggregation, thereby helping to maintain these molecules in monomeric, biologically active forms [15]. Furthermore, the hydrophobic character of the isopropyl and benzyl fragments was considered important for the intended applications. In the context of enzyme inhibition, the catalytic gorges of AChE and BChE have been described as deep, hydrophobic pockets. The lipophilic 4-(isopropylbenzyl)oxy groups may facilitate more favorable host–guest interactions within these enzymatic sites, potentially increasing binding affinity through hydrophobic interactions [16]. Selection of the central metal ions—indium (In), zinc (Zn), copper (Cu), cobalt (Co), and manganese (Mn)—was guided by the aim of investigating how coordination geometries and electronic configurations influence biological activity. Zinc was used as a standard diamagnetic reference due to its established stability and prevalence in medicinal inorganic chemistry [17]. Inclusion of indium was considered noteworthy because, relative to 3d transition metals, its larger atomic radius is often associated with pentacoordination and an out-of-plane geometry. This deviation from planarity may lead to spatial orientations that better accommodate structural features of AChE and BChE active sites, thereby providing a possible rationale for the low IC_50_ values observed for InPc, particularly against BChE. The transition metals copper, cobalt, and manganese were selected to evaluate the effects of paramagnetic centers and redox activity. Copper(II) complexes have been reported to interact with biomolecules, including through DNA intercalation and the generation of reactive oxygen species (ROS), which may be associated with the high antiproliferative activity observed in the MCF-7 cancer cell line [18]. Cobalt and manganese centers, in contrast, may provide variable oxidation states and potential axial coordination with amino acid residues (e.g., histidine or cysteine) within enzyme catalytic sites, thereby influencing inhibition behavior and mechanism [19]. By systematically varying these metal centers, a clearer understanding was sought of how the electronic environment of the Pc core may modulate antibacterial and anticancer responses. Motivated by these considerations, the enzyme inhibitory activity and the antibacterial and anticancer potential of a highly soluble 4-(isopropylbenzyl)oxy-substituted phthalonitrile ligand and its metal Pcs were examined.

## 2. Materials and methods

### 2.1. Synthesis and characterization

Details of the equipment, materials, and synthetic procedures were provided in the supporting information.

### 2.2. Anticancer activities

#### 2.2.1. Cell culture

MCF-7 human breast cancer cells were cultured in RPMI-1640 medium (Serena) supplemented with 10% fetal bovine serum (Capricorn; FBS-HI-11A), 100 U/mL penicillin, and 100 μg/mL streptomycin. Cells were incubated at 37 °C in a humidified atmosphere containing 5% CO_2_.

#### 2.2.2. In vitro cytotoxicity

MCF-7 cells were seeded at 7500 cells/well in 96-well plates containing RPMI-1640 medium and were allowed to adhere for 24 h. After incubation, the medium was removed, and the test compounds (dinitrile ligand, InPc, ZnPc, CuPc, CoPc, and MnPc) were applied to the cells at final concentrations of 1–30 μM. Dimethyl sulfoxide (DMSO) was used as the vehicle control. After 24 h, the culture medium was removed, and MTT reagent (5 mg/mL; 3-(4,5-dimethylthiazol-2-yl)-2,5-diphenyltetrazolium bromide) was added to each well. After 4 h of incubation, the MTT-containing medium was removed, and 100 μL DMSO was added to each well to dissolve the formazan crystals. Absorbance was recorded at 540 nm using a microplate spectrophotometer (Thermo Multiskan Go; Thermo Fisher Scientific, Waltham, MA, USA), and the number of viable cells was calculated according to equation [20]. The IC_50_ value (half-maximal inhibitory concentration) for each compound was determined by plotting compound concentration against the number of viable cells. All experiments were performed in three independent replicates.

#### 2.2.3. Examination of cell morphology

MCF-7 cells were seeded at 5 × 10^4^ per well in 24-well plates and were allowed to adhere for 24 h. After incubation, the medium was removed, and two compounds (CuPc and MnPc) were applied to the cells at 1.7 μM. In the control group, cells were incubated in RPMI-1640 medium. After 24 h of incubation, changes in cell morphology were examined by bright-field microscopy using an Axio microscope (Carl Zeiss AG, Oberkochen, Germany) [21].

### 2.3. Effect on acetylcholinesterase

The inhibitory activity of the synthesized compounds was assessed in vitro using an adapted Ellman method. The assay mixture was prepared by adding 100 μL of 1 M Tris–HCl buffer (pH 8.0) and a 10 mM DTNB solution as the chromogenic reagent. Distilled water and the test-compound solution, together with 10 μL of AChE, were added to the reaction mixture to achieve the desired final volume. The mixture was preincubated at 37 °C for 10 min. Subsequently, 10 mM acetylthiocholine iodide was added to initiate the enzymatic reaction. AChE catalyzes the hydrolysis of acetylthiocholine to thiocholine and acetate. In the absence of inhibitors, thiocholine reacts with DTNB to form the yellow-colored 5-thio-2-nitrobenzoic acid, which was detected by measuring absorbance at 412 nm [22]. Absorbance readings for both the control and sample cuvettes were recorded at the 5-min mark of the reaction. The inhibitory effects of the synthesized compounds on AChE were evaluated, and IC_50_ and K_i_ values were calculated from the experimental data.

### 2.4. Effect on butyrylcholinesterase

The inhibitory effects of the synthesized molecules on BChE were also evaluated. A procedure similar to that used for the AChE assay was followed; however, butyrylthiocholine iodide (the BChE substrate) was used in place of acetylthiocholine iodide.

### 2.5. Antibacterial activities

In this study, the antibacterial activity of the synthesized compounds was quantified using the disk diffusion method [23]. All test microorganisms were obtained from the Central Research Laboratory of Gümüşhane University, and antibacterial testing was conducted in the Food Engineering Laboratory. To determine antibacterial activity, bacterial strains representing were employed, encompassing both gram-positive (*Enterococcus faecalis* ATCC 29212, *Staphylococcus aureus* ATCC 25923, *Streptococcus pyogenes* ATCC 19615, *Bacillus subtilis* ATCC 6633, *Bacillus cereus* ATCC 9634) and gram-negative (*Salmonella typhimurium* ATCC 23566, *Escherichia coli* O157:H7 35150, *Klebsiella pneumoniae* ATCC 13883, *Escherichia coli* ATCC 25922, *Proteus vulgaris* ATCC 13315, and *Pseudomonas aeruginosa* ATCC 27853) organisms were used. A aliquot of 20 μL of the test solution (1 mg/mL in DMSO) was applied to sterile disks placed on nutrient agar, and the Petri dishes were incubated at 36 °C for 24 h. After incubation, inhibition-zone diameters were measured using a digital caliper. DMSO was employed as a negative control in the analysis. All antibacterial assays were performed in duplicate. The obtained data were subjected to Duncan’s multiple range test using SPSS Statistics 22 (IBM Corp., Armonk, NY, USA).

### 2.6. ADME calculations

In this study, the SwissADME platform was used to predict the ADME (absorption, distribution, metabolism, and excretion) properties of the target ligand and its related metal phthalocyanines (1–6) [24,25].

## 3. Results

### 3.1. Synthesis and optical spectra of the phthalocyanines

**Figure 1:**
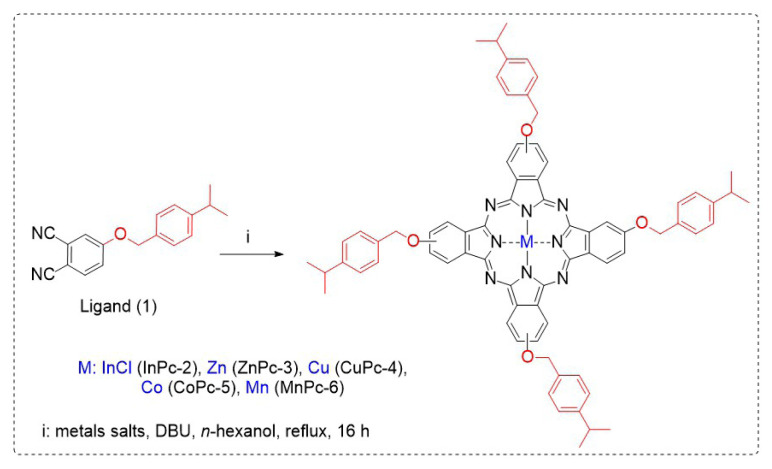
Molecular structures and synthetic pathways of the compounds.

The dinitrile compound 4-((4-isopropylbenzyl)oxy)phthalonitrile (1) and its indium(III) chloride, zinc(II), and copper(II) phthalocyanine (Pc) derivatives (2–4) were prepared according to reported procedures [26–28]. Cobalt and manganese Pcs were prepared for the first time in this study. Figure 1 shows the synthetic pathways and molecular structures of the ligand and its metal Pc derivatives.

FT-IR spectra of the synthesized cobalt(II) (CoPc) and manganese(III) (MnPc) Pcs were recorded to confirm formation of the macrocyclic structure and to characterize functional groups in the complexes. The characteristic vibrational frequencies are presented in Figures S1 and S2. Complete disappearance of the sharp absorption band at approximately 2230 cm^−1^, assigned to the C≡N stretching vibration of the phthalonitrile precursor (1), provided primary evidence for successful cyclotetramerization and formation of the metal Pc complexes. In the high-frequency region, weak aromatic C–H stretching bands were observed at 3056 cm^−1^ for CoPc and 3058 cm^−1^ for MnPc. Intense bands in the 2960–2850 cm^−1^ region were assigned to aliphatic C–H stretching vibrations (asymmetric and symmetric methyl/methylene modes) arising from isopropyl groups on the peripheral benzyloxy substituents. Formation of the phthalocyanine core was further supported by intense bands in the 1610–1450 cm^−1^ region, including peaks at 1609 cm^−1^ (CoPc) and 1606 cm^−1^ (MnPc), which were attributed to aromatic C=C skeletal vibrations and C=N stretching of the isoindole ring system. Furthermore, the presence of the (4-isopropylbenzyl)oxy substituents was supported by the distinct ether bands. Aromatic ether (Ar–O–C) bands were observed at 1228 and 1092 cm^−1^ for CoPc and at 1227 and 1080 cm^−1^ for MnPc. Bands observed in the lower-frequency region (around 746 cm^−1^) were ascribed to out-of-plane C–H deformation vibrations of substituted aromatic rings. Slight shifts in wavenumbers between CoPc and MnPc may suggest an influence of the central metal ion on the electron density of the macrocyclic ring system. The ^1^H NMR spectra of CoPc (5) and MnPc (6) could not be recorded due to the paramagnetic nature of the central metal ions. The presence of unpaired electrons in these metal centers induces rapid nuclear relaxation, resulting in severe line broadening that renders the signals uninterpretable. The molecular structures of CoPc (5) and MnPc (6) were further supported by MALDI-TOF mass spectrometry. The mass spectra of the CoPc (5) and MnPc (6) exhibited molecular ion peaks consistent with their calculated exact mass values. For CoPc, the experimental peak at m/z 1186.213 was attributed to the sodiated adduct [M+Na]^+^, which is consistent with the calculated neutral exact mass of 1163.44. Similarly, MnPc displayed a prominent peak at m/z 1160.943, which was assigned to the protonated molecular ion [M+H]^+^ and was consistent with the calculated neutral mass of 1159.44. Overall, these results provide strong evidence for successful formation of the target Pc structures (Figures S3 and S4).

**Figure 2:**
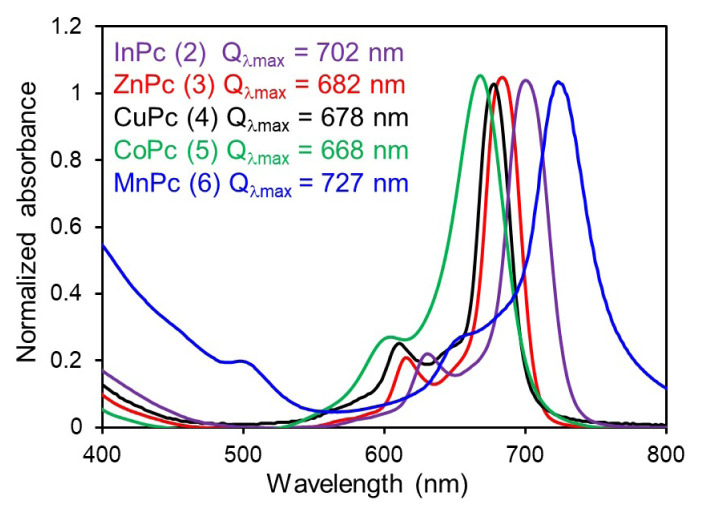
UV–Vis normalized absorption spectra of metal phthalocyanines (2–6).

Characteristic absorption bands are exhibited by Pcs in the UV–Vis spectrum due to their planar structures and extended π-conjugation. The primary absorption bands, typically observed around 600–700 nm (Q-band region) and 300–400 nm (Soret-band region), arise from electronic transitions within the molecule, mainly π-π* transitions [29]. UV–Vis absorption spectra were recorded in tetrahydrofuran (THF), and the intense, sharp, and well-defined Q-band maximum was centered at 702 nm for 2, 682 nm for 3, 678 nm for 4, 668 nm for 5, and 727 nm for 6 (Figure 2). These variations can be attributed to the dependence of Q-band shifts on the characteristics of the central metal ion, including ionic radius, coordination geometry, and electronic coupling between metal d orbitals and the Pc π system. The most prominent bathochromic (red) shift was observed for the indium Pc (InPc) derivative relative to the more planar zinc (ZnPc) and copper (CuPc) complexes. This phenomenon is commonly attributed to the large ionic radius of In^3+^ (approximately 80 pm), which exceeds the central cavity size of the Pc ring. Consequently, the indium ion is often described as adopting a pentacoordinated geometry and sitting significantly out of the macrocyclic plane. This displacement may induce a doming distortion of the Pc ring, which can lower D_4h_ symmetry and narrow the HOMO–LUMO energy gap. Destabilization of the a_1u_ orbital relative to the e_g_ orbital, ascribed to geometric strain, may result in the characteristic shift toward longer wavelengths [29]. Furthermore, the absorption characteristics of the manganese Pc (MnPc) complex showed a distinct red shift and band broadening, which may be explained by partially filled d orbitals at the transition-metal center. In MnPc systems, energetic proximity between manganese d orbitals and ligand p orbitals may allow significant orbital hybridization. This overlap may facilitate metal-to-ligand charge transfer or ligand-to-metal charge transfer transitions, which can be superimposed on the traditional π → π* transitions. Back-bonding from the manganese center may stabilize the LUMO of the macrocycle, thereby reducing the excitation energy required for the Q-band transition. In contrast, the zinc (ZnPc) and copper (CuPc) complexes exhibited more blue-shifted (hypsochromic) Q bands relative to InPc and MnPc. This observation is consistent with closed-shell (Zn) or nearly closed-shell electronic configurations and with a better fit within the central cavity, thereby maintaining predominantly planar D_4h_ symmetry. Overall, these results indicate that the optical properties of the synthesized complexes are sensitive to the coordination environment and the electronic nature of the metal center, thereby providing a rationale for the spectral variations observed in Figure 2.

### 3.2. Enzyme inhibition activity

Table 1 summarizes the IC_50_ values for inhibition of AChE and BChE by the synthesized Pcs. The results indicate that all synthesized compounds inhibited both enzymes to varying degrees; among the Pcs, CoPc and InPc showed the lowest IC_50_ values against AChE (Table 1). For BChE, higher IC_50_ values than those for AChE were observed, indicating that these compounds were generally more selective for AChE inhibition. Among the tested compounds, the lowest IC_50_ value against AChE was observed for CoPc (14.81 nM), closely followed by InPc (14.91 nM), whereas the lowest IC_50_ value against BChE was observed for InPc (56.35 nM) (Table 1). The IC_50_ value of usnic acid for AChE inhibition was reported as 1.27 **μg**/mL [30], whereas the IC_50_ value of rosmarinic acid against AChE was reported as 36.73 nM [31]. The K_i_ values and the type of inhibition for each compound are summarized in Table 2. The majority of compounds exhibited competitive inhibition; the lowest K_i_ value against AChE was observed for CuPc (3.08 nM), whereas the lowest K_i_ value against BChE was observed for CuPc (25.98 ± 1.97 nM) (Table 2). These results, indicating inhibition of both AChE and BChE, may support a dual-target approach for addressing cholinergic deficits associated with AD.

When the inhibition results for AChE and BChE were examined, all synthesized compounds were found to inhibit both enzymes to varying degrees. However, based on the IC_50_ and K_i_ values, higher selectivity toward AChE was observed. This is particularly important because AChE inhibition is widely recognized as relevant to cholinergic dysfunction in Alzheimer’s disease. The observation that InPc showed strong inhibition—particularly against BChE (IC_50_= 56.35 nM)—and that AChE inhibition was also pronounced for CoPc and InPc (IC_50_ = 14.81 and 14.91 nM, respectively) suggests that the electronic properties of the central metal ion may influence enzyme–ligand interactions.

When the K_i_ values and inhibition types were examined, it was determined that the majority of compounds acted through a competitive inhibition mechanism. This finding suggests that phthalocyanines may bind at or near the enzyme active site in a manner that competes with the substrate. In particular, the low K_i_ value obtained for CuPc against AChE (3.08 nM) suggests a strong interaction with the enzyme active site. These results indicate that the synthesized phthalocyanines exhibit strong inhibitory activity relative to naturally occurring AChE inhibitors such as usnic acid [30] and rosmarinic acid [31] reported in the literature.

In this context, simultaneous inhibition of AChE and BChE may offer a dual-target approach for addressing cholinergic deficits associated with Alzheimer’s disease. In a study of phthalocyanine derivatives, metal phthalocyanines were reported to exert strong inhibitory effects on AChE and BChE. In particular, a previous study on triazole-substituted phthalocyanines reported an IC_50_ value of approximately 40 nM for AChE [32]. In the present study, lower IC_50_ values against AChE (14.81–18.39 nM) were observed for metal-containing phthalocyanines such as InPc, CuPc, and CoPc, suggesting that the central metal ion may strengthen enzyme–ligand interactions. This observation may be attributed to structural features of the AChE active site that allow more favorable interactions with the phthalocyanine core and peripheral substituents. Furthermore, the low K_i_ values obtained in this study and the predominantly observed competitive inhibition pattern support the hypothesis of binding at or near the enzyme active site, consistent with competition with the substrate. In summary, relative to metal-free or differently substituted phthalocyanines reported in the literature, the metal phthalocyanines presented in this study may have higher potential—particularly with respect to AChE inhibition—and may be considered candidate inhibitors relevant to cholinergic mechanisms in Alzheimer’s disease.

### 3.3. Antimicrobial properties

The antibacterial activity of the synthesized compounds against selected gram-positive and gram-negative bacterial strains is presented in Table 3.

Compound 4 exhibited the most pronounced inhibitory effect against *Enterococcus faecalis* ATCC 29212 (p < 0.001), whereas compounds 2, 3, and 4 showed statistically similar inhibitory effects against this strain. Compound 2 exhibited a more pronounced inhibitory effect against *Staphylococcus aureus* ATCC 25923 than the other compounds (p < 0.05). Nevertheless, the inhibitory effects of compounds 3 and 5 against this strain were statistically similar. Regarding *Streptococcus pyogenes* ATCC 19615, only compounds 2 and 5 exhibited inhibitory effects, with compound 2 showing a more pronounced effect (p < 0.001). The greatest effect against *Bacillus subtilis* ATCC 6633 was observed for compound 3. The inhibitory effect of compound 3 was statistically similar to those of compounds 2 and 4. For *Bacillus cereus* ATCC 9634, the inhibition-zone diameter produced by compound 4 was statistically similar to that of the control. Thus, compound 4 was not found to be effective against *Bacillus cereus* ATCC 9634 under the tested conditions. Furthermore, no inhibitory effect was observed for compound 6 against the selected gram-positive bacteria.

Compound 1 exhibited the most pronounced inhibitory effect against *Salmonella typhimurium* ATCC 23566 among all tested compounds (p < 0.01). Furthermore, no inhibitory effect was observed for compounds 2 and 6 against *Salmonella typhimurium* ATCC 23566. Nevertheless, the inhibitory effects of the control and compound 4 against *Salmonella typhimurium* ATCC 23566 were statistically indistinguishable. Accordingly, compound 4 was not found to exert measurable antibacterial activity against *Salmonella typhimurium* ATCC 23566 under the tested conditions.

Similarly, compound 1 exhibited a notable inhibitory effect against *Escherichia coli* O157:H7 35150 (p < 0.05), whereas compounds 2 and 4 showed statistically similar inhibitory activity. The greatest inhibitory effect against *Escherichia coli* ATCC 25922 was observed for compound 2 (p < 0.001). Furthermore, the inhibitory effects of compounds 1 and 5 against *Escherichia coli* ATCC 25922 were statistically similar. Whereas compounds 1 and 6 showed similar inhibitory effects against *Klebsiella pneumoniae* ATCC 13883, compound 2 exhibited the most pronounced effect (p < 0.001). The inhibition-zone diameters pronounced by compound 5 were statistically similar to those of the control. Accordingly, compound 5 was not found to exhibit measurable antibacterial activity against *Klebsiella pneumoniae* ATCC 13883 relative to the control under the tested conditions. Compound 5 was not found to be effective against *Proteus vulgaris* ATCC 13315, whereas compound 3 exhibited the most pronounced effect (p < 0.001). Nevertheless, the inhibitory effects of the other compounds against *Proteus vulgaris* ATCC 13315 were statistically similar. No inhibitory effect was observed for compound 6 against *Pseudomonas aeruginosa* ATCC 27853. Compound 3 exhibited the most pronounced inhibitory effect against *Pseudomonas aeruginosa* ATCC 27853 (p < 0.001), whereas the inhibitory effects of compounds 2 and 4 were statistically similar. In contrast, the inhibition-zone diameter produced by compound 1 against *Pseudomonas aeruginosa* ATCC 27853 did not differ significantly from that of the control. In light of these findings, compound 1 was not found to exhibit measurable antibacterial activity against *Pseudomonas aeruginosa* ATCC 27853 under the tested conditions.

### 3.4. Anticancer activities

The cytotoxic effects of the compounds (ligand, InPc, ZnPc, CuPc, CoPc, and MnPc) were evaluated using a colorimetric MTT assay. The IC_50_ values of compounds are presented in Table 4. All compounds exhibited antiproliferative activity at low micromolar concentrations. In addition, differences in cytotoxicity were observed among the phthalocyanines. The IC_50_ values ranged from 1.7 to 4.2 μM. Higher antiproliferative activity was observed for CuPc than for the other compounds. The ligand (1) was found to be less cytotoxic than the phthalocyanines (2–6). To date, the anticancer activities of various phthalocyanines have been evaluated in different cancer cell lines, including breast and lung cancer models. Although some phthalocyanine derivatives have been reported to exhibit therapeutic effects [33], the cytotoxicity results for thiophenol-containing ZnPc against lung cancer (A549) and breast cancer (MCF-7) cells were similar to those obtained in the present study [34,35]. The observed IC_50_ values (1.7–4.2 μM) were comparable to, or lower than, those reported for several metal phthalocyanines tested under similar conditions, suggesting promising antiproliferative potential for the present compounds.

**Figure 3:**
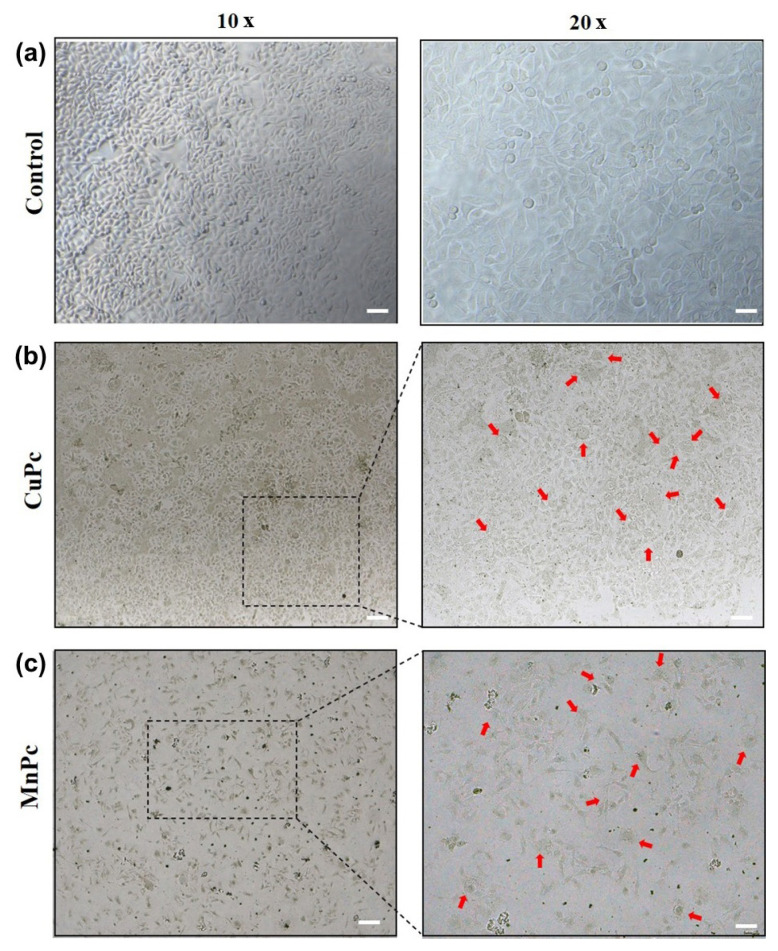
Bright-field images of MCF-7 cells after 24 h of treatment with CuPc (b) or MnPc (c) (1.7 μM), acquired at 10× and 20× magnification. Scale bar: 200 μm.

Changes in cell morphology were examined by bright-field microscopy. MCF-7 cells exhibit an epithelial-like morphology; these relatively large cells typically contain more than one nucleolus per cell [36]. In the control group, the cells retained their regular shape and membrane integrity (Figure 3a). However, after 24 h of treatment with CuPc and MnPc (1.7 μM), observable morphological changes were noted. CuPc- and MnPc-treated cells showed enlarged, irregular shapes with indistinct membrane borders (Figures 3b and 3c). Irregular, enlarged cells are indicated by red arrows in the images (20× magnification). It was hypothesized that treatment with CuPc and MnPc may lead to molecular and phenotypic changes accompanied by morphological alterations in cell shape and appearance.

Selection of the metals (In, Zn, Cu, Co, and Mn) was based on their distinct electronic, redox, and coordination properties, which may influence the biological behavior of phthalocyanines. Specifically, these metals can adopt different oxidation states, redox behaviors, and coordination geometries, thereby enabling comparative evaluation of structure–activity relationships within a single ligand framework [37]. Among the synthesized compounds, the strongest antiproliferative activity in the MTT assay was observed for CuPc and MnPc. Because morphological analysis is primarily qualitative and supportive rather than a quantitative screening method, microscopy was intentionally focused on the most biologically active compounds. This approach is consistent with previous reports, in which morphological evaluations are typically performed for the most potent candidates to provide mechanistic insight while avoiding redundancy [38,39]. Furthermore, CuPc and MnPc have previously been associated with cytotoxic effects in cancer cells, inducing ROS production, apoptosis/necrosis, and cell-death–associated morphological changes [39,40], consistent with the morphological observations in the present study (Figure 3). These findings further supported selection of CuPc and MnPc for morphological examination.

### 3.5. In silico ADME and drug-likeness predictions

In this study, the pharmacokinetic properties and drug-similarity profiles of the synthesized compounds 1–6 were evaluated using the SwissADME online platform. The calculated physicochemical parameters included molecular weight, lipophilicity indices based on different logP approaches, topological polar surface area (TPSA), and estimated aqueous solubility, which provide preliminary information on the absorption and distribution potential of the compounds (Table 5).

According to SwissADME predictions, all compounds were predicted to have high gastrointestinal absorption and to be capable of crossing the blood–brain barrier. In addition, because none of the compounds were predicted to be *p*-glycoprotein substrates, the risk that active efflux may limit bioavailability was considered to be low. Drug-similarity assessments were performed based on the Lipinski, Ghose, Veber, Egan, and Muegge criteria, and all compounds were found to comply with these rules [40–44].

However, all ADME results presented here are predictions derived from QSAR-based statistical models and represent an early-stage in silico screen. No density functional theory (DFT) or ab initio level electronic structure calculations were performed within the scope of this study. Therefore, these pharmacokinetic predictions should be validated by experimental studies or more advanced computational methods. In this context, the present study focuses on computational ADME approaches for evaluating the early-stage pharmaceutical suitability of candidate compounds rather than on quantum-chemical calculations (Table 5).

## 4. Conclusion

In summary, the enzyme inhibitory activity and the antibacterial and anticancer potential of a highly soluble phthalonitrile derivative (1) and its metal phthalocyanines (Pcs; indium, zinc, copper, cobalt, and manganese; 2–6) were investigated. The results showed that antibacterial activity varied among compounds depending on the bacterial species tested. These findings suggest that the compounds may have potential as antibacterial agents against the tested pathogens. The synthesized compounds exhibited inhibitory activity against both AChE and BChE, with strong potency observed for several Pcs, particularly InPc and CuPc. In addition, high cytotoxicity was observed against the MCF-7 breast cancer cell line. However, in vivo studies are needed to validate these findings and to further assess potential clinical applicability and anticancer activity. These findings suggest that the compounds may be considered candidate inhibitors relevant to cholinergic mechanisms associated with AD.

## Supporting information

### 1. Materials and Equipment

All reagents and solvents were of reagent-grade quality and were obtained from commercial suppliers. The homogeneity of the products was tested in each step by TLC. The solvents were stored over molecular sieves. All solvents were dried and purified as described by Perrin and Armarego [1]. IR spectra were recorded on a Perkin Elmer Spectrum One FT-IR (ATR sampling accessory) spectrophotometer, and electronic spectra on a Shimadzu UV-1280 and Shimadzu 1800 UV-Vis spectrophotometer. Mass analyses were recorded on a Bruker MALDI-TOF (Matrix-Assisted Laser Desorption/Ionization-Time-Of-Flight mass, Rheinstetten, Germany) spectrometer using alpha-cyano-4-hydroxy-cinnamic acid and dithranol (DIT) as matrix materials.

### 2. Synthesis

A mixture of 4-((4-isopropylbenzyl)oxy)phthalonitrile (**1**) (0.100 g, 0.36 mmol), anhydrous CoCl_2_, MnCl_2_ ~(0.030 g, excess) in *n*-hexanol and 1,8-diazabicyclo[5.4.0]un- dec-7-ene (DBU, 0.05 mL) were heated to reflux temperature in a sealed tube under an argon atmosphere for 16 h. After the reaction was completed, the resulting green mixture was cooled to room temperature. The crude products were precipitated by methanol-water, filtered off, and then washed with the methanol-water mixture. The crude products were purified by column chromatography using a mixture of CH_2_CI_2_ and EtOH (5:2 v/v) as the eluent. Obtained Pcs were highly soluble in ethyl acetate, CH_2_CI_2_, and CHCl_3_. Yield of **5**: 49 mg (46.8%). FT-IR (υ_max_/cm^−1^): 3062 (m, Ar. C-H), 2950–2868 (Aliph. -C-H), 1609 (m, Ar. -C=C), 1477 (m, Aliph. -C-H), 1348 (s, Ar.–C-N), 1228 (s, -C-O-C). UV-Vis λ_max_ (nm) THF: 696, 334. Anal. calc. for C_72_H_64_CoN_8_O_4_: C, 74.28; H, 5.54; Co, 5.06; N, 9.62; O, 5.50. Found: C, 74.14; H, 5.33; N, 9.86. MS (MALDI-TOF): *m/z* 1186.21 [M+Na]^+^. Yield of **6**: 31 mg (31.1%). FT-IR (υ_max_/cm^−1^): 3062 (m, Ar. C-H), 2950–2868 (Aliph. -C-H), 1606 (m, Ar. -C=C), 1477 (m, Aliph. -C-H), 1348 (s, Ar.–C-N), 1228 (s, -C-O-C). UV-Vis λ_max_ (nm) THF: 751, 523, 321. Anal. calc. for C_72_H_64_MnN_8_O_4_: C, 74.53; H, 5.56; Mn, 4.73; N, 9.66; O, 5.52. Found: C, 74.34; H, 5.43; N, 9.96. MS (MALDI-TOF): *m/z* 1160.9 [M+H]^+^.

### 3. Characterization

Figure S1 FT-IR spectrum of cobalt phthalocyanine (**5**).

Figure S2 FT-IR spectrum of manganese phthalocyanine (**6**).

Figure S3 MALDI-TOF mass spectrum of cobalt phthalocyanine (**5**).

Figure S4 MALDI-TOF mass spectrum of manganese phthalocyanine (**6**).

References1

PerrinAWLFDD

Purification of Laboratory Chemicals
2nd ed
Pergamon P
1989


## Figures and Tables

**Table 1:** Comparison of IC_50_ values for inhibition of AChE and BChE by the synthesized compounds.

Compound ID	AChE		BChE	
	IC_50_ (nM)	R^2^	IC_50_ (nM)	R^2^
Ligand (1)	65.39	0.9952	256.72	0.9920
InPc (2)	14.91	0.9916	56.35	0.9932
ZnPc (3)	18.39	0.9931	64.18	0.9943
CuPc (4)	16.46	0.9909	63.59	0.9973
CoPc (5)	14.81	0.9887	65.39	0.9963
MnPc (6)	15.82	0.9886	64.18	0.9903
THA	192.54	0.9924	123.78	0.9917

**Table 2:** Comparison of K_i_ values for inhibition of AChE and BChE by the synthesized compounds.

Compound ID	AChE		BChE	
	K_i_ (nM)	Type of inhibition	K_i_ (nM)	Type of inhibition
Ligand (1)	19.13 ± 1.70	Competitive	163.73 ± 14.17	Noncompetitive
InPc (2)	3.70 ± 0.10	Competitive	36.46 ± 0.92	Competitive
ZnPc (3)	3.45 ± 0.09	Competitive	33.10 ± 0.74	Competitive
CuPc (4)	3.08 ± 1.12	Competitive	54.55 ± 1.71	Noncompetitive
CoPc (5)	3.47 ± 0.19	Competitive	28.33 ± 4.54	Competitive
MnPc (6)	3.18 ± 0.25	Competitive	25.98 ± 1.97	Competitive
THA	27.95 ± 4.09	Competitive	120.01 ± 25.96	Competitive

**Table 3:** Antibacterial activity of the synthesized compounds against selected gram-positive and gram-negative bacterial strains.

Bacteria strains		Compounds						ANOVA(p)	
	Control	1	2	3	4	5	6		
**Gram-positive**	*Enterococcus faecali*s ATCC 29212	9.13 ± 0.25^ab^	9.97 ± 0.47abc	10.63 ± 0.82^c^	10.07 ± 0.75^c^	10.80 ± 1.21^c^	N.D	N.D	*0.000*
	*Staphylococcus aureus* ATCC 25923	9.93 ± 0.15abc	10.53 ± 0.05^c^	10.57 ± 0.29^b^	10.40 ± 0.36^bc^	N.D	10.30 ± 1.03^bc^	N.D	*0.014*
	*Streptococcus pyogenes* ATCC 19615	6.00 ± 0.00^a^	N.D	9.63 ± 0.05^c^	N.D	N.D	8.37 ± 0.45^b^	N.D	*0.000*
	*Bacillus subtilis* ATCC 6633	6.00 ± 0.00^a^	N.D	9.70 ± 0.10^c^	9.77 ± 0.32^c^	9.23 ± 0.30^c^	8.53 ± 0.80^b^	N.D	*0.000*
	*Bacillus cereus* ATCC 9634	6.00 ± 0.00^a^	N.D	N.D	N.D	7.40 ± 0.30^a^	N.D	N.D	*0.000*
**Gram-negative**	*Salmonella typhimurium* ATCC 23566	9.40 ± 0.10abc	10.70 ± 0.34^d^	N.D	10.40 ± 0.36^cd^	9.60 ± 0.27abc	10.13 ± 1.20bcd	N.D	*0.008*
	*Escherichia coli* O157:H7 35150	9.20 ± 0.10^a^	10.60 ± 0.60^b^	10.50 ± 0.52^b^	9.93 ± 0.32^ab^	10.37 ± 0.68^b^	9.90 ± 0.44^ab^	9.80 ± 0.27^ab^	*0.028*
	*Escherichia coli* ATCC25922	7.10 ± 0.10^a^	8.60 ± 0.27^d^	9.23 ± 0.25^e^	8.00 ± 0.10^bc^	7.87 ± 0.15^b^	8.60 ± 0.52^d^	8.37 ± 0.05^cd^	*0.000*
	*Klebsiella pneumoniae* ATCC 13883	8.27 ± 0.15^ab^	9.30 ± 0.76^c^	10.67 ± 0.20^d^	N.D	9.03 ± 0.12^bc^	8.43 ± 0.20^ab^	9.40 ± 0.72^c^	*0.000*
	*Proteus vulgaris* ATCC 13315	8.03 ± 0.35^a^	8.63 ± 0.80^ab^	8.77 ± 0.64^ab^	9.70 ± 0.50^b^	8.67 ± 1.48^ab^	N.D	8.57 ± 0.20^ab^	*0.000*
	*Pseudomonas aeruginosa* ATCC 27853	8.67 ± 0.45^ab^	9.03 ± 0.72^ab^	9.40 ± 1.30abc	10.30 ± 0.36^c^	9.27 ± 0.46abc	9.83 ± 0.15^bc^	N.D	*0.048*

a–d Mean values sharing different superscript letters within the same row differ significantly at p < 0.05.

N.D: not detected.

**Table 4:** Cytotoxic effects of compounds 1–6, as determined by the MTT assay, against the MCF-7 breast cancer cell line.

Compound ID	IC_50_ value (μM)
Ligand (1)	7.5 ± 2.05
InPc (2)	3.7 ± 0.2
ZnPc (3)	4.0 ± 0.6
CuPc (4)	1.7 ± 0.25
CoPc (5)	4.2 ± 0.15
MnPc (6)	2.8 ± 0.35

**Table 5:** ADME-related parameters of compounds 1–6.

Properties	Parameters	Compound ID					
		1	2	3	4	5	6
Physicochemical properties	Molecular weight	276.33	1255.60	1170.71	1168.88	1164.26	1160.27
	Molar refractivity	81.43	363.38	357.53	357.53	357.53	357.53
	TPSA	56.81	120.94	120.94	120.94	120.94	120.94
Lipophilicity	iLOGP	3.00	3.00	3.00	3.00	3.00	3.00
	XLOGP3	3.95	3.95	3.95	3.95	3.95	3.95
	WLOGP	3.98	13.46	12.77	12.77	12.77	12.77
	MLOGP	2.63	2.63	2.63	2.63	2.63	2.63
	SILICOS-IT	4.50	4.50	4.50	4.50	4.50	4.50
	Consensus log Po/w	3.61	3.61	3.61	3.61	3.61	3.61
Water solubility	log S (ESOL)	−4.20	−4.20	−4.20	−4.20	−4.20	−4.20
	log S (Ali)	−4.84	−4.84	−4.84	−4.84	−4.84	−4.84
	log S (SILICOS-IT)	−6.05	−6.05	−6.05	−6.05	−6.05	−6.05
Pharmacokinetics	GI absorption	High	High	High	High	High	High
	BBB permeant	Yes	Yes	Yes	Yes	Yes	Yes
	P-gp substrate	No	No	No	No	No	No
	CYP1A2 inhibitor	Yes	Yes	Yes	Yes	Yes	Yes
	CYP2C19 inhibitor	Yes	Yes	Yes	Yes	Yes	Yes
	CYP2C9 inhibitor	No	No	No	No	No	No
	CYP2D6 inhibitor	No	No	No	No	No	No
	CYP3A4 inhibitor	Yes	Yes	Yes	Yes	Yes	Yes
	log Kp (skin permeation)	−5.18	−5.18	−5.18	−5.18	−5.18	−5.18
Drug-likeness	Lipinski	Yes	Yes	Yes	Yes	Yes	Yes
	Ghose	Yes	Yes	Yes	Yes	Yes	Yes
	Veber	Yes	Yes	Yes	Yes	Yes	Yes
	Egan	Yes	Yes	Yes	Yes	Yes	Yes
	Muegge	Yes	Yes	Yes	Yes	Yes	Yes
	Bioavailability score	0.55	0.55	0.55	0.55	0.55	0.55
Medicinal chemistry	PAINS	0 alert	0 alert	0 alert	0 alert	0 alert	0 alert
	Brenk	0 alert	0 alert	0 alert	0 alert	0 alert	0 alert
	Lead-likeness	1 violation: XLOGP3 > 3.5	1 violation: XLOGP3 > 3.5	1 violation: XLOGP3 > 3.5	1 violation: XLOGP3 > 3.5	1 violation: XLOGP3 > 3.5	1 violation: XLOGP3 > 3.5
	SA score	2.23	2.23	2.23	2.23	2.23	2.23

## References

[1] Perrin AWLFDD Purification of Laboratory Chemicals 2nd ed Pergamon P 1989

