# Supplementary figures and images for: Investigation of the enzyme-inhibitory, antibacterial, and anticancer properties of metal phthalocyanines
Source: Turk J Chem. 2026 Feb 16;50(2):173–85. doi: 10.55730/1300-0527.3788 (PMC13189370; doi:10.55730/1300-0527.3788)

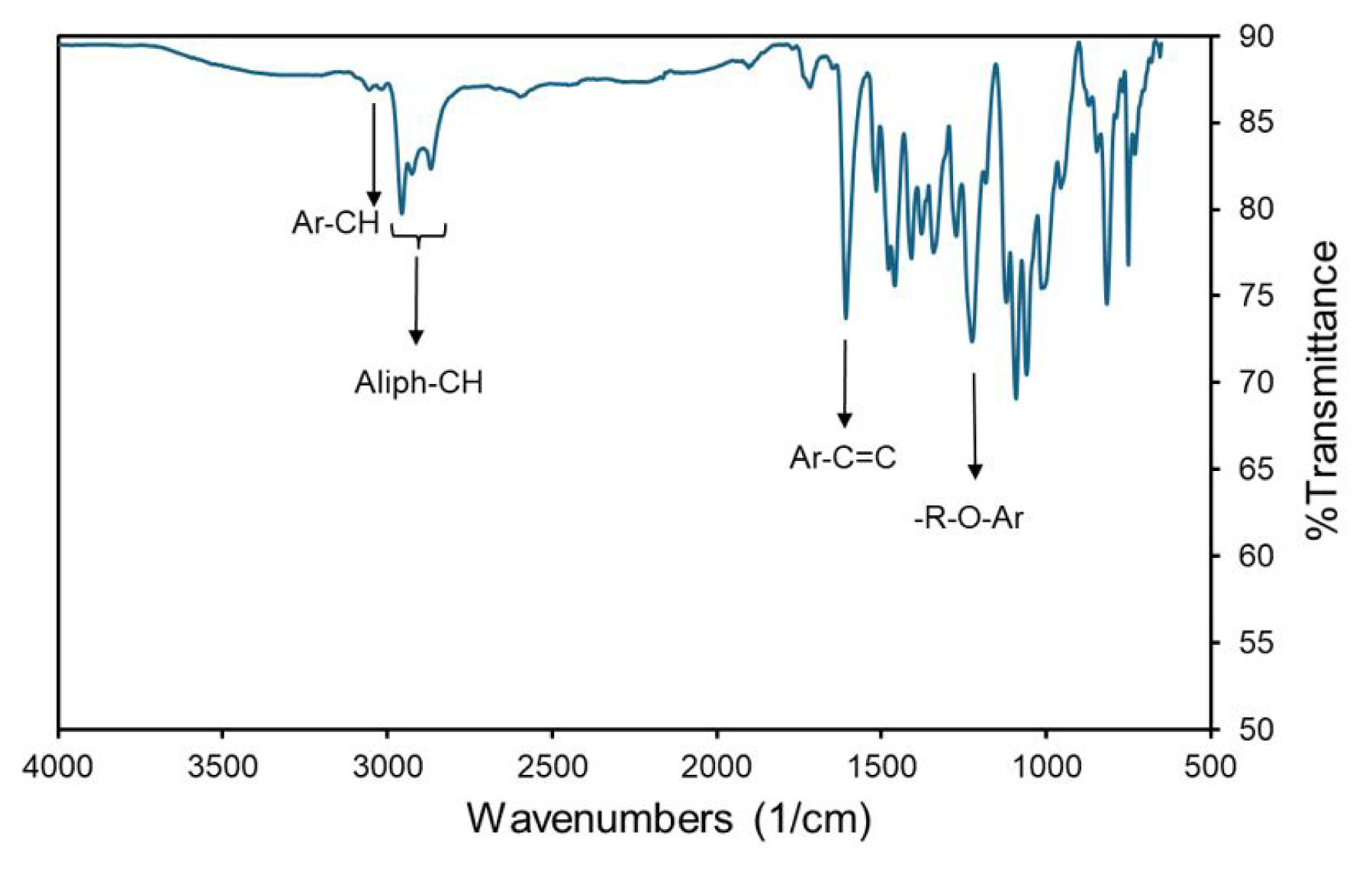

Supplement: Figure S1 — FT-IR spectrum of cobalt phthalocyanine (5). [file tjc-50-02-173s1.tif]

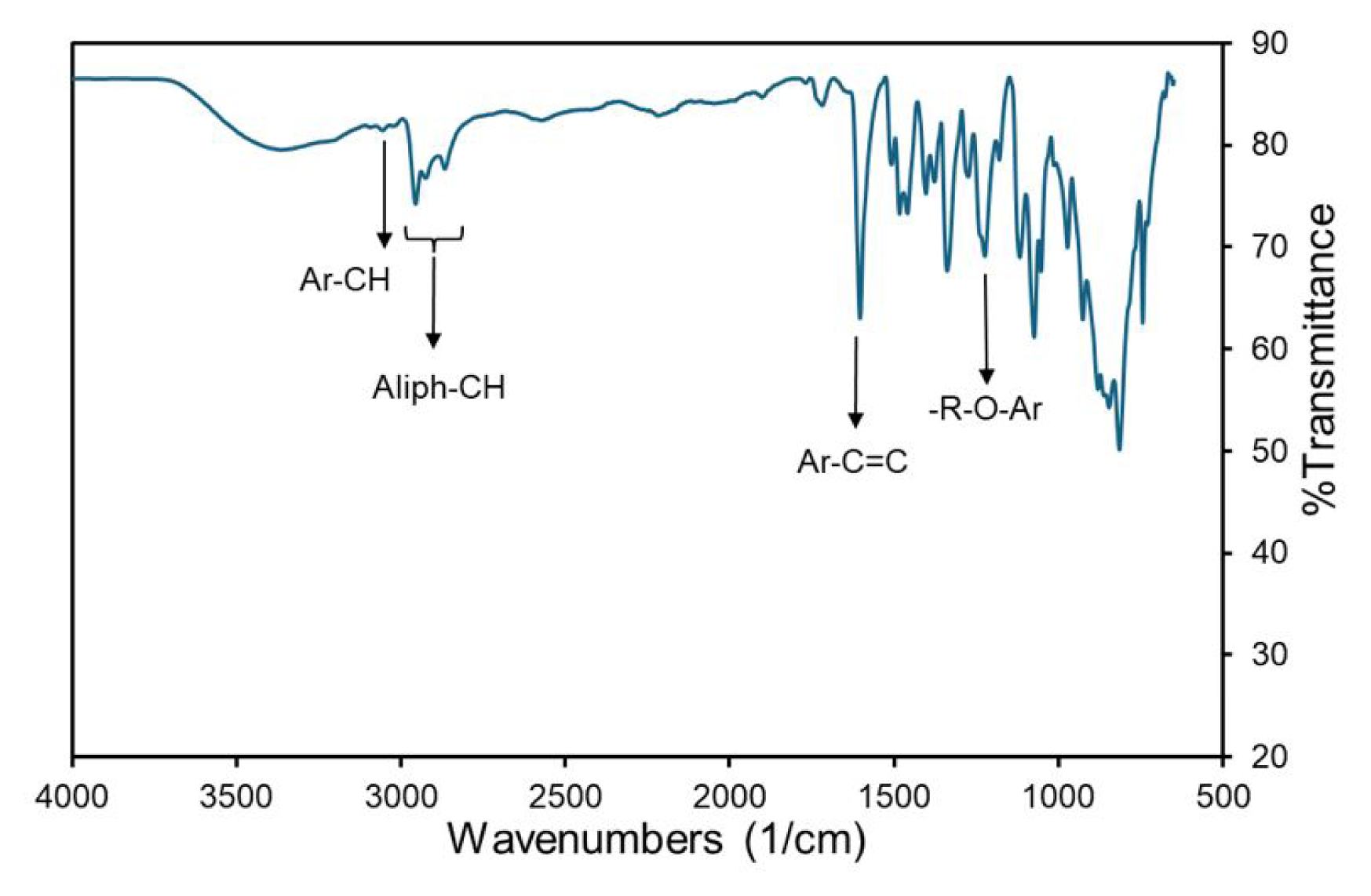

Supplement: Figure S2 — FT-IR spectrum of manganese phthalocyanine (6). [file tjc-50-02-173s2.tif]

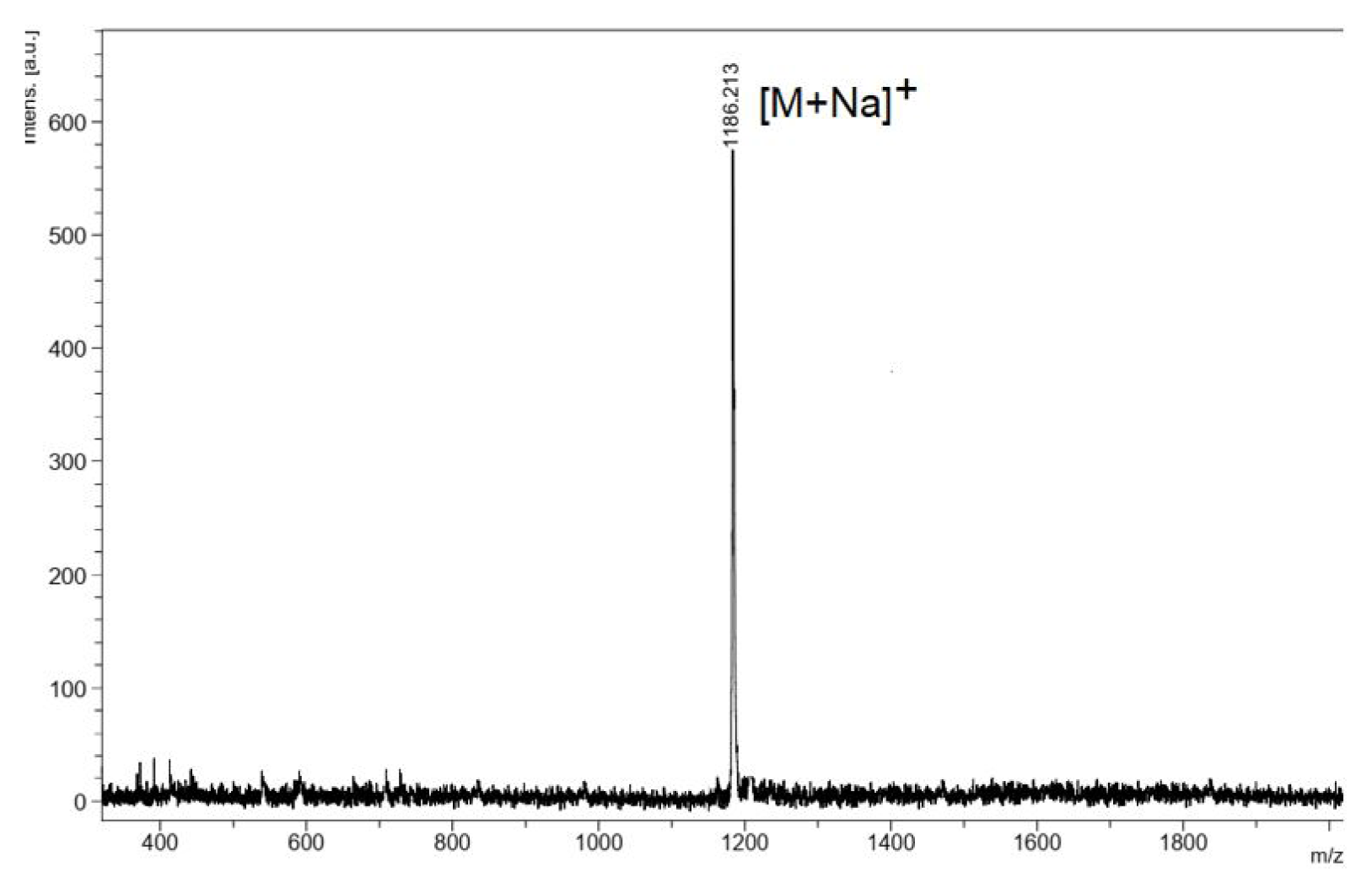

Supplement: Figure S3 — MALDI-TOF mass spectrum of cobalt phthalocyanine (5). [file tjc-50-02-173s3.tif]

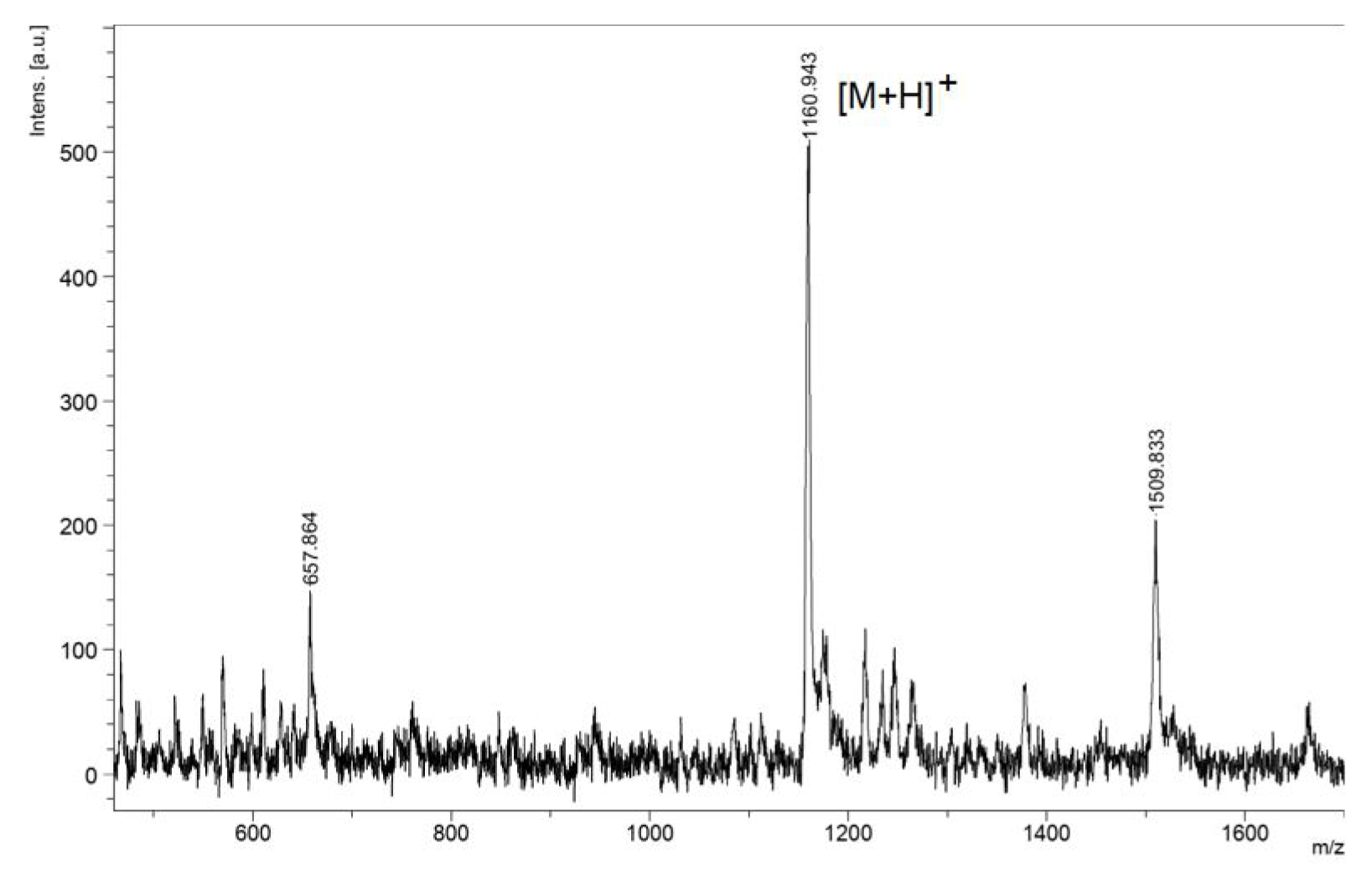

Supplement: Figure S4 — MALDI-TOF mass spectrum of manganese phthalocyanine (6). [file tjc-50-02-173s4.tif]
